# Supplementary material for: Noval insights and therapeutic strategies for tumor-induced kidney pathologies
Source: J Exp Clin Cancer Res. 2024 Oct 19;43:289. doi: 10.1186/s13046-024-03205-6 (PMC11490039; doi:10.1186/s13046-024-03205-6)
Supplement: Supplementary file 1 — Supplementary Material 1 [file 13046_2024_3205_MOESM1_ESM.docx]

Noval Insights and Therapeutic Strategies for Tumor-Induced Kidney Pathologies

Meng Wang^1^, Yong Han^1^, Chao Zhang^2^*

*^1^Department of Endocrinology, Songjiang Research Institute, Songjiang Hospital Affiliated to Shanghai Jiao Tong University School of Medicine, Shanghai, 200011, China*

*^2^Department of Orthopedics and Precision Research Center for Refractory Diseases, Shanghai General Hospital,* *Shanghai Jiao Tong University School of Medicine, Shanghai, 200080, China*

*Corresponding author:

Chao Zhang, [zhangchao@shsmu.edu.cn](mailto:zhangchao@shsmu.edu.cn), ORCID: 0000-0001-6418-8370

## Keywords

Acute kidney injury；Cancer；Hormone；GPCR；Single-cell sequencing

## Abstract

Recent progress in elucidating the role of specific antidiuretic hormones in Drosophila models has provided valuable insights into the mechanisms underlying tumor-induced renal dysfunction. Xu et al. identified the mammalian neurokinin 3 receptor (TACR3), a homolog of the G protein-coupled receptor TkR99D in fruit flies, as a potential therapeutic target for alleviating renal tubular dysfunction in mice with malignant neoplasms[1]. Here, we commented on these findings by emphasizing the structural and evolutionary significance of TACR3 and provided an in-depth analysis of cell type specific expression of TACR3 in response to renal injury and expressional dynamics during renal carcinoma progression. The implications of these findings for transforming the therapeutic approaches to renal complications associated with oncological disorders were highlighted. We also acknowledged the limitations of current experimental models in this study and emphasized the necessary clinical validation in the future. These insights could contribute to the advancement of diagnostic and therapeutic strategies for treating tumor-related renal pathologies.

**Take Home Message:**

1. Recently, the neurokinin 3 receptor (TACR3) has emerged as a crucial factor of renal dysfunction among cancer patients, suggesting its potential as a target for innovative therapeutic intervention.
2. Our analysis delves into the evolutionary insights of TACR3 from multiple studies on both Drosophila and mammals.
3. We elucidate the role of TACR3 in renal pathologies by integrating multi-omics datasets and conducting a thorough phylogenetic and structural analyses.

## Damage in cancer patients

Renal damage has emerged as a notable complication of cancer patients. The pathogenesis is multifactorial, involving direct mechanical effects on the kidney, such as tissue compression or invasion, systemic metabolic alterations, and indirect impacts via bioactive mediators such as cytokines and hormones[2]. Renal damage is also often precipitated by standard treatments such as chemotherapy and radiotherapy, known for their nephrotoxic potential[3, 4]. These complex interactions pose a significant challenge for oncological management, necessitating treatment strategies that effectively balance tumor suppression and renal preservation.

## Identification of novel antidiuretic hormone and receptor

In this groundbreaking study, Xu et al. identified ITPF (see Glossary), a novel antidiuretic hormone in Drosophila, as a key regulator of cancer-associated renal dysfunction[1]. They revealed that tumor-derived ITPF impaired renal function, manifesting in abdominal bloating and fluid accumulation. This dysfunction was mediated by targeting G protein-coupled receptors (GPCRs) in renal stellate cells, specifically in the Malpighian tubules, that led to the activation of cGMP cascade and subsequent inhibition of fluid excretion. Significantly, the study has drawn a parallel illustration with mammals by identifying the neurokinin 3 receptor (TACR3) as the mammalian counterpart of the Drosophila receptor, which highlighted TACR3 as a potential therapeutic target for treating cancer-related renal dysfunction in human patients.

The agonists of TACR3 include natural peptide Neurokinin B (NKB) and Substance P, as well as the synthetic senktide. Recent study using cryogenic electron microscopy has provided a fine conformational insights on how TACR3 is activated by these peptides[5]. Those results revealed non-canonical activation and specific interaction within extracellular regions of TACR3. Upon activation, TACR3 triggers downstream signaling cascades mediated by G proteins and lead to various intracellular responses, that includes Gonadotropin-Releasing Hormone (GnRH) regulation[6], neurotransmitter release[7], and alterations in intracellular calcium concentrations[8]. Consequently, TACR3 is primarily recognized for its vital role in controlling the reproductive axis[9, 10], mood[11, 12], pain response[13], and body temperature[14].

## Evolutionary perspective of TACR3

From a systemic evolutionary perspective, the TACR3 gene exhibits a notable evolutionary trajectory, evidenced by its presence across a diverse array of species, including both invertebrates and mammals (Figure 1). Comparative sequence alignment demonstrates substantial homology of TACR3 protein, particularly in pivotal regions for maintaining pharmacological and physiological functions. Particularly the conserved transmembrane domains that are integral for ligand binding and signal transduction, play a crucial role for the proper interaction of TACR3 with neurokinin B, and ensure the intracellular signaling cascades[15]. Furthermore, the sequence similarity extends to the conserved function across various species. In teleost, TACR3-like receptors imply potential participation in reproduction processes analogous to those observed in mammals[16]. The cross-species functional conservation underscores the evolutionary significance of TACR3 in maintaining vital biological functions.

## Therapeutic potential of TACR3 antagonists

TACR3 antagonists have shown potential therapeutic benefits across a variety of human disorders. Table 1 provides a detailed comparison of current drug development pipelines targeting TACR3. The recent FDA approval of the first TACR3 antagonist for managing menopausal hot flushes represents a precedent and a significant advance of clinical therapy for targeting TACR3[17]. Beyond the initial application, recent evidence proposes the targeting of TACR3 as a novel anti-angiogenic strategy for oncological interventions. In vitro studies with human umbilical vein endothelial cells (HUVECs) have demonstrated the antagonism of TACR3 in inhibiting cellular migration[18]. This finding is critical for elucidating the endogenous function of TACR3 for tumor angiogenesis and metastasis with a highlight of its clinical potential for treating multiple human diseases. Xu et al. assessed the therapeutic effects of Fezolinetant and Pavinetant on renal dysfunction across various tumor models, including those of gastric, lung, colon, and melanoma, as well as in patient-derived xenograft (PDX) models[1]. This study provides a foundation of TACR3 antagonism for transition into clinical treatment for renal impairments caused by tumors. However, we also noticed that they did not directly compare the efficacy of Fezolinetant and Pavinetant across these tumor types.

Clinical evidence of several TACR3 antagonists have emphasized the requirement for hepatic monitoring due to asymptomatic increases in hepatic transaminases, though bilirubin levels remain unaffected[19]. Additionally, recent studies indicated minor increase of blood glucose level resulted from TACR3 antagonism[20]. Fezolinetant showed common side effects in 2.3%–4.3% of participants in a 52-week safety trial. These included abdominal pain, diarrhea, insomnia, back pain, hot flashes, and elevated hepatic transaminases, with incidences notably higher than those observed in the placebo group[20]. Although TACR3 antagonism show promise for cancer treatment, particularly in tumor-associated disorders, these adverse effects emphasize the importance of ongoing hepatic monitoring and further evaluation to ensure the safe application in conjunction with other means of cancer therapies.

## Structural insights into TACR3 activation

GPCRs, including TACR3, have emerged as significant targets for cancer treatment due to their essential roles for regulating abnormal cell growth and facilitating tumor invasion and metastasis[21]. However, drug development pipeline targeting TACR3 faces challenges similar to those of other GPCRs, such as low specificity and high toxicity, which limit their clinical application[22]. Identifying the precise binding sites and conformational changes upon binding of antagonist is crucial for the structure-based drug design of TACR3[23]. Despite the well-accepted importance, the correlation between the TACR3-antagonist complex and its inhibitory mechanism remains poorly understood. To bridge this gap, future research should aim to gain a deep conformational illustration of TACR3 complex with advanced structural techniques, such as Cryo-Electron Microscopy (Cryo-EM). Here, a molecular docking platform was employed to delineate the binding topography of TACR3 with its inhibitors, Fezolinetant and Pavinetant, as depicted in Figure 2A-B. The interactive locus of Fezolinetant is identified within the fourth ECL and fifth transmembrane helices of TACR3, while Pavinetant is postulated to predominantly interact within the first transmembrane helix. These findings suggest that these domains are integral to ligand selectivity and instrumental in modulating antagonistic signal transduction pathway of the receptor. Elucidation of these binding sites has enhanced our understanding of the pharmacological properties of TACR3 and will facilitate the rational design of targeted therapeutic molecules in the future.

## Opportunities of drug development for TACR3

Furthermore, an interactive network analysis of TACR3 could reveal new biomarkers for early diagnosis and disease monitoring[24, 25]. A network diagram illustrating the known interactive protein partners of TACR3 is depicted in Figure 2C. Researchers could utilize new technologies, such as single-cell RNAseq to further identify notable regulators of TACR3 membrane expression and downstream signaling pathways, with a potential to facilitate the TACR3 targeted development of combined therapeutic approaches. To overcome the challenges associated with small molecule screening of TACR3, recruitment of a combination of advanced models and technologies in future studies is highly recommended. These novel approaches should commence with the artificial intelligence (AI) and virtual screening platforms for the primary identification of potential molecular candidates. Subsequently, organoid tumor models could be employed for a comprehensive high-throughput in vitro assessment of drug efficacy. This strategy aims not only to discover small molecule inhibitors but also to develop peptide-based nanobodies and proteolysis-targeting chimeras (PROTAC) that could specifically target TACR3. The subsequent phase should utilize murine models to evaluate the in vivo safety and toxicity of the lead compounds. Such comprehensive approach integrates advanced computational and experimental methodologies to enhance the efficiency and efficacy of the drug development progress of TACR3 (Figure 2D).

In a preceding study, genetic analyses demonstrated that TACR3 inhibitors significantly decreased the likelihood of ischemic heart disease in the male population, proposing an innovative preventive measure against this condition[26]. Concurrently, the preliminary evidence showing a concurrent reduction in prostate cancer risk with TACR3 antagonism underscores the necessity for in-depth biological and clinical investigation to confirm these findings in the near future. In our extensive analysis of the roles of TACR3 across various cancer types, we expanded our examination beyond the primary model in the study by Xu et al., which included partial digestion tumors such as ApcMin/+ colon tumors, ipMFC mouse gastric tumors, ipHCT human colon tumors, PDX patient-derived gastric xenografts, and LLC mouse lung tumors. The previous focus restricted the application of these findings to a broad range of cancer types.

## Comprehensive analysis of TACR3 expression

To obtain a more comprehensive understanding of TACR3 across various tumors, we integrated an extensive array of multi-omics datasets, including single-cell transcriptomics, spatial transcriptomics and TCGA(Figure 3). Our findings uncovered a dynamic Tacr3 expression pattern in mouse models of ischemia-reperfusion injury (IRI) at various time points, as evidenced by the t-SNE visualization and bubble chart of transcriptomic profiles from the IRI kidney. This expression varied significantly over time, that indicated a potential role of Tacr3 in renal injury and recovery processes. Moreover, spatial expressional distribution emphasized the roles of Tacr3 in specific kidney regions upon injury, that highlighted its localized function in renal repair mechanism. Furthermore, comparison of transcriptional levels of TACR3 in various renal cell types during acute kidney injury to normal conditions revealed a unique expression profile and implicated the participation of TACR3 in the pathology of kidney injury. Crucially, our analysis included human datasets and examined the expression levels of TACR3 across various stages of kidney renal clear cell carcinoma (KIRC) and kidney renal papillary cell carcinoma (KIRP). The violin plots showed significant differential TACR3 expression between different tumor stages and statistical analysis further confirmed the significance of this variance. Insights from our multi-omics analysis suggest a significant role of TACR3 not only in renal injury but also in the tumorigenesis and progression of renal carcinomas.

## Concluding remarks

In summary, Xu et al identifies a novel pathway involving antidiuretic hormone, and underscores its impact on cancer-induced renal dysfunction. However, the precise molecular mechanism of this pathway remain elusive. The expanding role of TACR3 antagonists, including Fezolinetant, is significant, ranging from managing menopausal symptoms to potential applications in hormone-related disorders and cancer therapy. Despite these promising findings, direct evidence from clinical trials or human studies is scarce, making the clinical relevance and therapeutic application speculative at this stage. Future research should concentrate on the conserved function and specificity of TACR3, Fezolinetant's binding sites, and the heterogeneity of TACR3 expression across various tumor types and cell models **(see Outstanding questions)**. This should be complemented by clinical trials to validate the safety and efficacy of these findings in human patients, thereby assessing their feasibility as potential therapeutic options. Finally, the future development of TACR3 antagonists could be advanced through innovative drug design and screening strategies.

## Outstanding questions

What is the precise molecular mechanism by which tumor-derived ITPF impairs renal function in cancer patients?

How does TACR3 interact with other receptors and affect downstream signaling pathways in the context of cancer-associated renal dysfunction?

Can TACR3 antagonists be safely and effectively integrated into current oncological treatment regimens to mitigate renal damage?

What kind of role does TACR3 play in the broader context of tumor angiogenesis and metastasis, and how can this be targeted therapeutically?

How does the expression of TACR3 vary across different stages and renal cancer types, and what implications does this have for medical intervention?

## References:

1. Xu, W., G. Li, Y. Chen, X. Ye, and W. Song, *A novel antidiuretic hormone governs tumour-induced renal dysfunction.* Nature, 2023. **624**(7991): p. 425-432.

2. Finkel, K.W. and J.R. Foringer, *Renal disease in patients with cancer.* Nat Clin Pract Nephrol, 2007. **3**(12): p. 669-78.

3. Claudel, S.E., M. Gandhi, A.B. Patel, and A. Verma, *Estimating kidney function in patients with cancer: A narrative review.* Acta Physiol (Oxf), 2023. **238**(2): p. e13977.

4. Aapro, M. and V. Launay-Vacher, *Importance of monitoring renal function in patients with cancer.* Cancer Treat Rev, 2012. **38**(3): p. 235-40.

5. Sun, W., F. Yang, H. Zhang, Q. Yuan, S. Ling, Y. Wang, et al., *Structural insights into neurokinin 3 receptor activation by endogenous and analogue peptide agonists.* Cell Discov, 2023. **9**(1): p. 66.

6. Topaloglu, A.K., F. Reimann, M. Guclu, A.S. Yalin, L.D. Kotan, K.M. Porter, et al., *TAC3 and TACR3 mutations in familial hypogonadotropic hypogonadism reveal a key role for Neurokinin B in the central control of reproduction.* Nat Genet, 2009. **41**(3): p. 354-358.

7. Skorupskaite, K., J.T. George, J.D. Veldhuis, and R.A. Anderson, *Neurokinin B Regulates Gonadotropin Secretion, Ovarian Follicle Growth, and the Timing of Ovulation in Healthy Women.* J Clin Endocrinol Metab, 2018. **103**(1): p. 95-104.

8. Pal, S., J. Wu, J.K. Murray, S.H. Gellman, M.A. Wozniak, P.J. Keely, et al., *An antiangiogenic neurokinin-B/thromboxane A2 regulatory axis.* J Cell Biol, 2006. **174**(7): p. 1047-58.

9. Cañete, H., I. Dorta, M. Hernández, A. Cejudo Roman, L. Candenas, F.M. Pinto, et al., *Differentially regulated expression of neurokinin B (NKB)/NK3 receptor system in uterine leiomyomata.* Hum Reprod, 2013. **28**(7): p. 1799-808.

10. Cejudo Roman, A., F.M. Pinto, I. Dorta, T.A. Almeida, M. Hernández, M. Illanes, M. Tena-Sempere, and L. Candenas, *Analysis of the expression of neurokinin B, kisspeptin, and their cognate receptors NK3R and KISS1R in the human female genital tract.* Fertil Steril, 2012. **97**(5): p. 1213-9.

11. Cui, W.Q., W.W. Zhang, T. Chen, Q. Li, F. Xu, Q.L. Mao-Ying, W.L. Mi, Y.Q. Wang, and Y.X. Chu, *Tacr3 in the lateral habenula differentially regulates orofacial allodynia and anxiety-like behaviors in a mouse model of trigeminal neuralgia.* Acta Neuropathol Commun, 2020. **8**(1): p. 44.

12. Wojtas, M.N., M. Diaz-González, N. Stavtseva, Y. Shoam, P. Verma, A. Buberman, et al., *Interplay between hippocampal TACR3 and systemic testosterone in regulating anxiety-associated synaptic plasticity.* Mol Psychiatry, 2023.

13. Zhang, W.W., T. Chen, S.Y. Li, X.Y. Wang, W.B. Liu, Y.Q. Wang, et al., *Tachykinin receptor 3 in the lateral habenula alleviates pain and anxiety comorbidity in mice.* Front Immunol, 2023. **14**: p. 1049739.

14. Rance, N.E., P.A. Dacks, M.A. Mittelman-Smith, A.A. Romanovsky, and S.J. Krajewski-Hall, *Modulation of body temperature and LH secretion by hypothalamic KNDy (kisspeptin, neurokinin B and dynorphin) neurons: a novel hypothesis on the mechanism of hot flushes.* Front Neuroendocrinol, 2013. **34**(3): p. 211-27.

15. Noel, S.D., A.P. Abreu, S. Xu, T. Muyide, E. Gianetti, C. Tusset, et al., *TACR3 mutations disrupt NK3R function through distinct mechanisms in GnRH-deficient patients.* Faseb j, 2014. **28**(4): p. 1924-37.

16. Zhou, W., S. Li, Y. Liu, X. Qi, H. Chen, C.H. Cheng, X. Liu, Y. Zhang, and H. Lin, *The evolution of tachykinin/tachykinin receptor (TAC/TACR) in vertebrates and molecular identification of the TAC3/TACR3 system in zebrafish (Danio rerio).* Mol Cell Endocrinol, 2012. **361**(1-2): p. 202-12.

17. Mullard, A., *FDA approves first-in-class NK3 receptor antagonist for hot flushes.* Nat Rev Drug Discov, 2023. **22**(7): p. 526.

18. Wang, T., S. Chen, S. Wang, L. Shi, C. Wang, J. Zhang, et al., *Targeting neurokinin-3 receptor: a novel anti-angiogenesis strategy for cancer treatment.* Oncotarget, 2017. **8**(25): p. 40713-40723.

19. Lee, A., *Fezolinetant: First Approval.* Drugs, 2023. **83**(12): p. 1137-1141.

20. Neal-Perry, G., A. Cano, S. Lederman, R.E. Nappi, N. Santoro, W. Wolfman, et al., *Safety of Fezolinetant for Vasomotor Symptoms Associated With Menopause: A Randomized Controlled Trial.* Obstet Gynecol, 2023. **141**(4): p. 737-747.

21. Liu, Y., S. An, R. Ward, Y. Yang, X.X. Guo, W. Li, and T.R. Xu, *G protein-coupled receptors as promising cancer targets.* Cancer Lett, 2016. **376**(2): p. 226-39.

22. Dowden, H. and J. Munro, *Trends in clinical success rates and therapeutic focus.* Nat Rev Drug Discov, 2019. **18**(7): p. 495-496.

23. Shukla, A.K., G. Singh, and E. Ghosh, *Emerging structural insights into biased GPCR signaling.* Trends Biochem Sci, 2014. **39**(12): p. 594-602.

24. Schmidlin, F., O. Déry, N.W. Bunnett, and E.F. Grady, *Heterologous regulation of trafficking and signaling of G protein-coupled receptors: beta-arrestin-dependent interactions between neurokinin receptors.* Proc Natl Acad Sci U S A, 2002. **99**(5): p. 3324-9.

25. Florido, A., E. Moreno, E.I. Canela, and R. Andero, *Nk3R blockade has sex-divergent effects on memory in mice.* Biol Sex Differ, 2022. **13**(1): p. 28.

26. Schooling, C.M., *Genetic validation of neurokinin 3 receptor antagonists for ischemic heart disease prevention in men - A one-sample Mendelian randomization study.* EBioMedicine, 2022. **77**: p. 103901.

27. Kirita, Y., H. Wu, K. Uchimura, P.C. Wilson, and B.D. Humphreys, *Cell profiling of mouse acute kidney injury reveals conserved cellular responses to injury.* Proc Natl Acad Sci U S A, 2020. **117**(27): p. 15874-15883.

28. Dixon, E.E., H. Wu, Y. Muto, P.C. Wilson, and B.D. Humphreys, *Spatially Resolved Transcriptomic Analysis of Acute Kidney Injury in a Female Murine Model.* J Am Soc Nephrol, 2022. **33**(2): p. 279-289.

## Glossary

**ITPF (Inhibitory Tumor Peptide Factor)**

A novel antidiuretic hormone identified in Drosophila, shown to impair renal function in cancer-associated renal dysfunction.

**G protein-coupled receptors (GPCRs)**

A large family of seven-transmembrane cell surface receptors that respond to various external signals and activate intracellular signaling pathways through the interaction with G proteins.

**TACR3 (Tachykinin Receptor 3)**

Also known as neurokinin 3 receptor (NK3R), a receptor involved in various physiological processes, including reproductive hormone release, mood regulation, and pain response.

**Cryo-electron microscopy (Cryo-EM)**

A microscopy technique that allows the imaging of samples at cryogenic temperatures, providing detailed structural conformation of biological molecules.

**GnRH (Gonadotropin-Releasing Hormone)**

A hormone responsible for the release of follicle-stimulating hormone and luteinizing hormone from the anterior pituitary gland, with a crucial role in regulating reproduction.

**HUVECs (Human Umbilical Vein Endothelial Cells)**

Endothelial cells derived from the vein of the umbilical cord, commonly used in studies of vascular biology and angiogenesis.

**KIRC (Kidney Renal Clear Cell Carcinoma)**

A type of kidney cancer characterized by the presence of clear cells in the tumor, often associated with mutations in the VHL gene.

**KIRP (Kidney Renal Papillary Cell Carcinoma)**

A type of kidney cancer that forms in the lining of the kidney tubules and is characterized by a papillary growth pattern.

**PROTAC (Proteolysis-Targeting Chimera)**

A type of molecule designed to target specific proteins for degradation by the proteasome, used in targeted protein degradation therapies.

**Single-cell sequencing**

A technique that allows the analysis of the genomic, transcriptomic, or epigenomic content of individual cells, providing insights into cellular heterogeneity and function.

## Declaration

### **Ethics approval and consent to participate** Not applicable.

### **Consent for publication** Not applicable.

### Availability of data and materials

All of the data generated or analyzed in this study are included in the manuscript.

### Competing interests

The authors declare that they have no conflict of interest.

### Funding

This work was supported by the National Science and Technology Major Project (Grant No.2023ZD0506801), National Natural Science Foundation of China (Grant No. 32271165 & 82400980) and Startup Foundation for Advanced Talents of Shanghai General Hospital of Shanghai Jiao Tong University School of Medicine.

### Authors' contributions

Investigation and concept establishment: CZ and MW; Writing‒original draft preparation: MW; Writing‒review and editing: YH and CZ; Supervision: CZ; Funding acquisition: CZ.

### Acknowledgements

# We appreciate the financial support by the National Natural Science Foundation of China and Startup Foundation for Advanced Talents of Shanghai General Hospital of Shanghai Jiao Tong University School of Medicine.Graphical Abstract


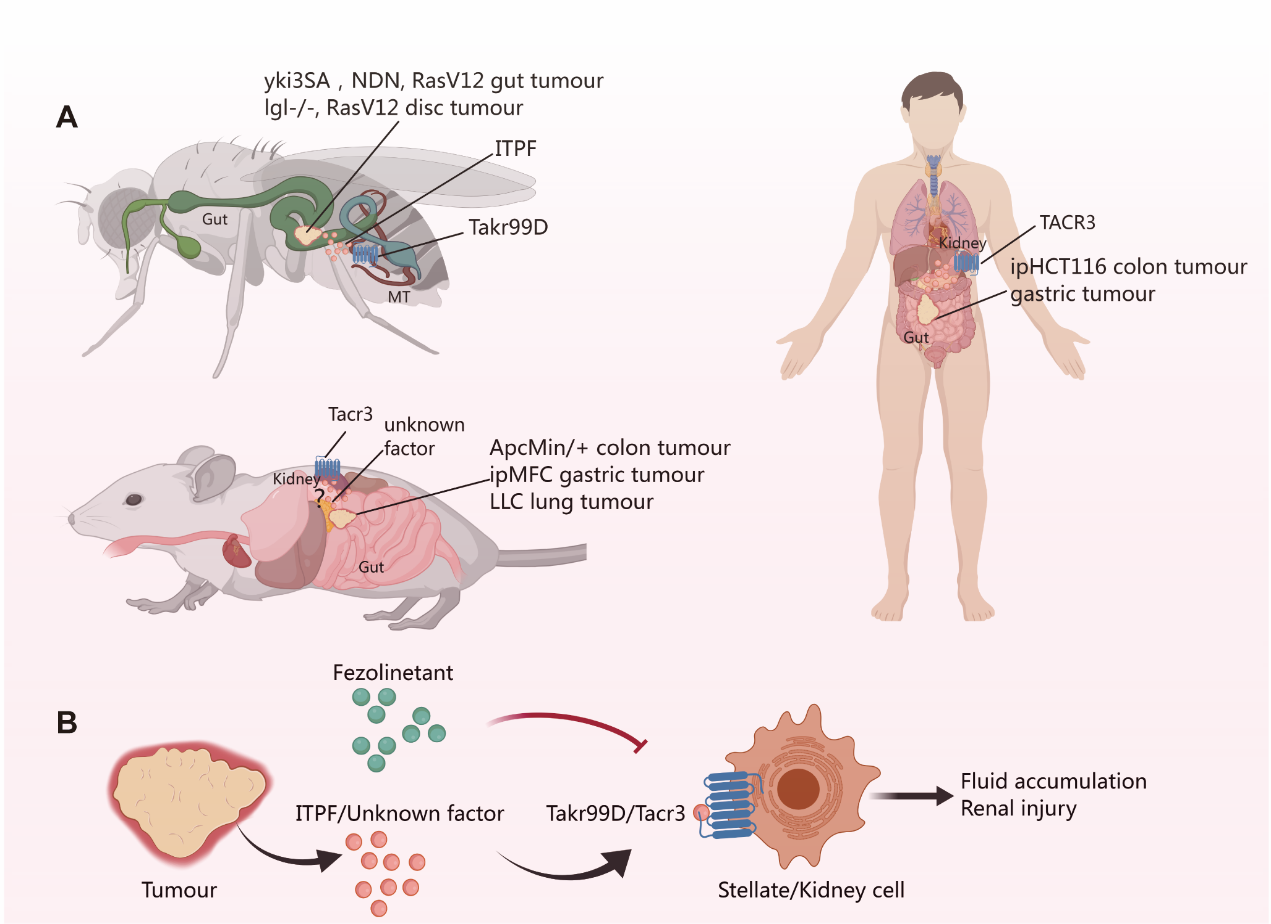


**Therapeutic Targeting of Tachykinin Receptors in Tumor-Induced Renal Injury Across Species in Oncological Models.**

# Figure legends


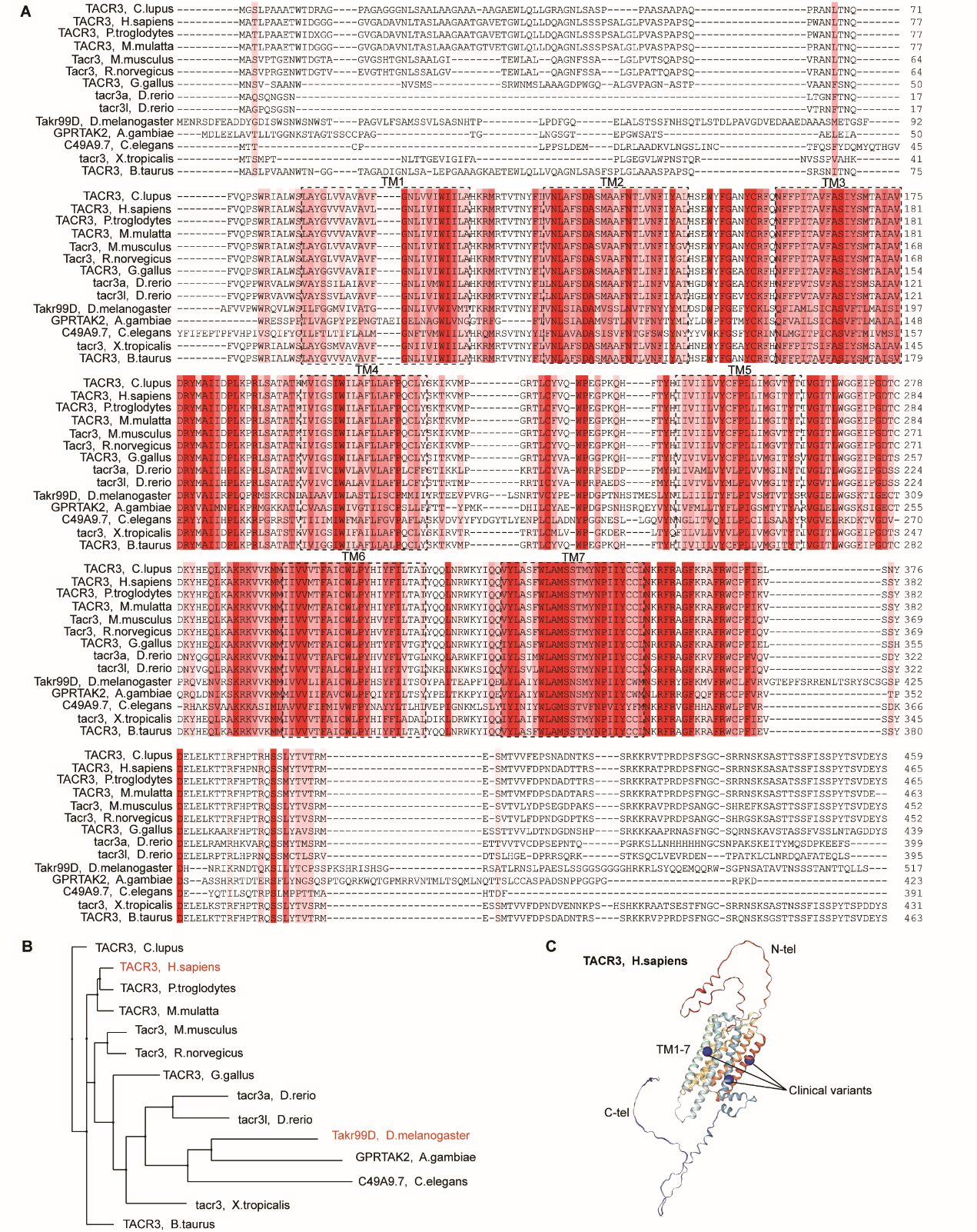


**Figure 1: Comparative Analysis and Structural Mapping of Neurokinin 3 Receptors of Multiple Species for Therapeutic Targeting.**

A) Comparative sequence alignment of neurokinin 3 receptors (TACR3) in various species. Highly conserved regions are shaded in red to indicate critical functional domains preserved from humans (H. sapiens) to birds (G. gallus) and fish (D. rerio). Drosophila melanogaster's TkR99D, Anopheles gambiae's GPRKTA2, and Caenorhabditis elegans' C49A7.7 demonstrate their conservation relative to mammalian TACR3s. Seven transmembrane regions are marked in black boxes.

B) Phylogenetic Tree showing the evolutionary relationships between the neurokinin 3 receptors (TACR3) of different species, rooted with the most divergent sequences. The branching pattern denotes evolutionary distances, positioning human TACR3 in relation to its counterparts from other mammals, birds, fish, and selected invertebrates.

C) 3D structural representation of human neurokinin 3 receptor (TACR3), with transmembrane regions TM1-TM7 shown in ribbon format. N-terminal (N-tel) and C-terminal (C-tel) ends are labeled, with clinical variants (blue) illustrating interactions with transmembrane regions (UniProt: P29371).


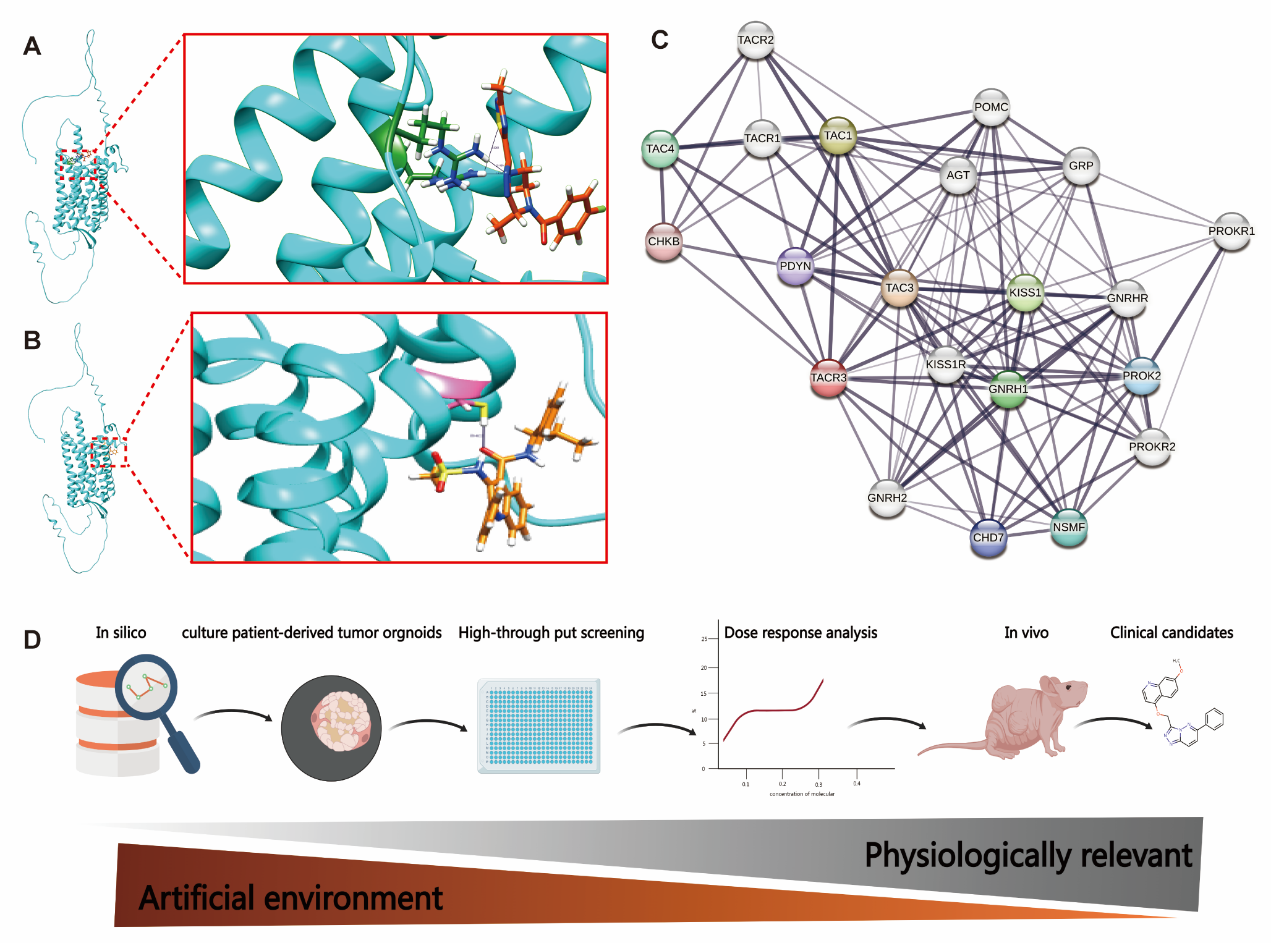


**Figure 2: Integrated Drug Discovery Approaches for TACR3 Antagonists.**

A-B) Predicted molecular interaction of Fezolinetant (A) and Pavinetant (B) with the TACR3 receptor, highlighting the ligand-receptor binding site.

C) Interactive network of TACR3 with related peptides and receptors, illustrating the complexity of TACR3-mediated signaling pathways.

D) Schematic representation of the drug discovery process for TACR3 targeting drugs, from in silico modeling to clinical candidate selection.


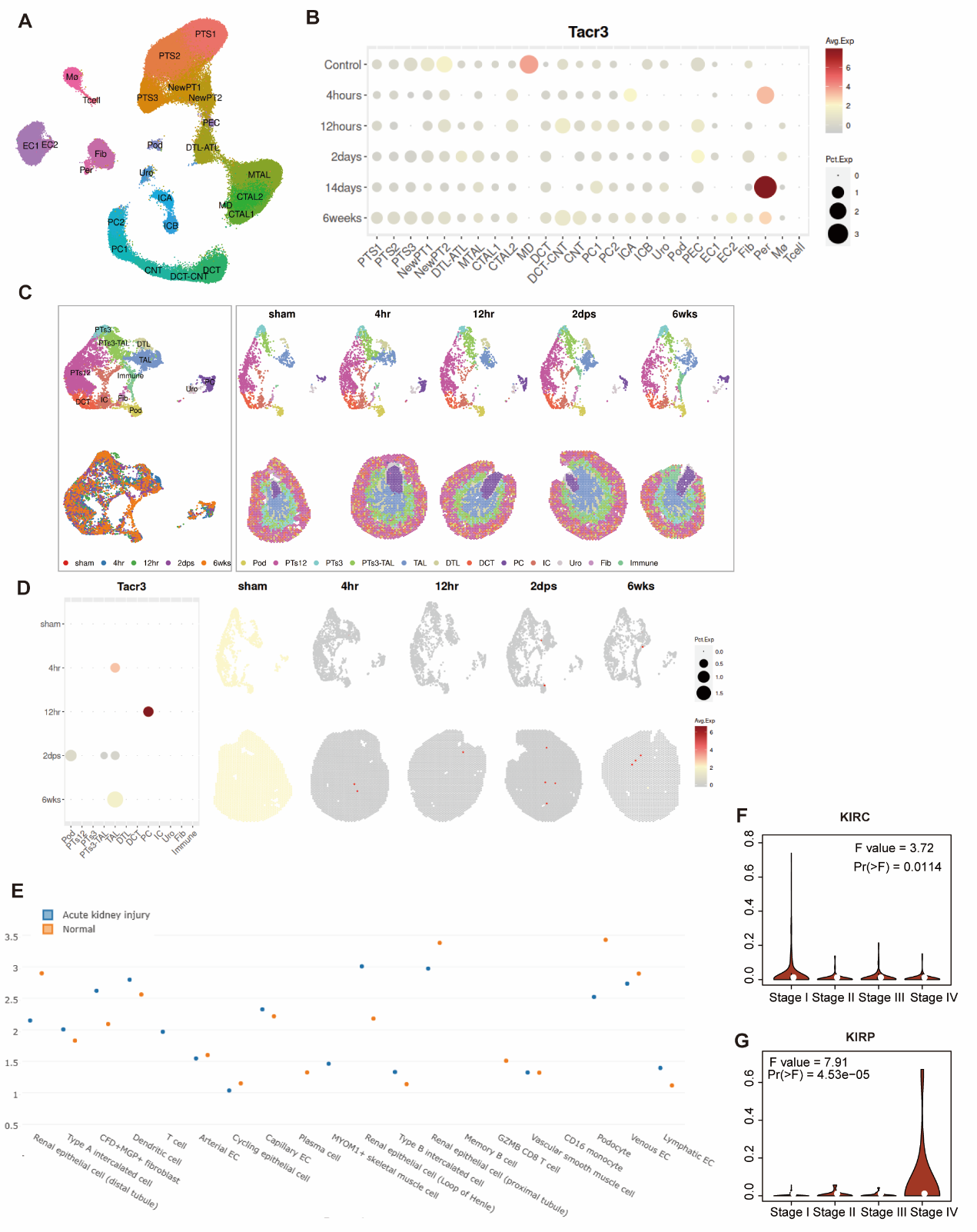


**Figure 3: Multi-Omics Analysis of TACR3 in Renal Injury and Tumorigenesis Across Murine and Human Models.**

A) t-SNE visualization of transcriptomic profiles in mouse IRI kidney at various time points (Sham, 4 hrs, 12 hrs, 2 days, 14 days, and 6 weeks). Data is sourced from Kirita et al, PNAS 2020[27].

B) Bubble chart illustration of Tacr3 expression across various renal cell types at different time points post-IRI (4 hours to 6 weeks). Color intensity and bubble size represent expression levels and statistical significance of changes, respectively. The data is sourced from Kirita et al, PNAS 2020[27].

C) t-SNE plots depicting cellular composition and gene expression changes in female Mouse IRI kidney at various time points (sham, 4 hours, 12 hours, 2 days, 6 weeks). The data is sourced from Dixon et al JASN 2021[28].
D) Spatial distribution of Tacr3 in the kidney across different stages post-injury. Red color intensity indicates Tacr3 expression levels in specific kidney regions over time.

E) Dot plot showing TACR3 expression levels in various renal cell types during acute kidney injury (AKI) compared to normal conditions. Color denotes condition (blue for AKI, orange for normal), and y-axis position indicates TACR3 expression levels.

F-G) Violin plot of TACR3 expression levels across different stages of kidney renal clear cell carcinoma (KIRC) and kidney renal papillary cell carcinoma (KIRP). F value and p-value denote the statistical significance of expression differences between stages.

**Table 1. Current Development Progress of TACR3 Antagonists for Clinical Indications.**

| **Name** | **DrugBank ID** | **Highest stage of development** | **Indications** |
| --- | --- | --- | --- |
| Fezolinetant | DB15669 | FDA Approved | Hot Flashes， Menopause, Renal Impairme, Hepatic Impairment, Vasomotor Symptoms |
| Pavinetant | DB11692 | Phase II | PCOS (polycystic ovary syndrome), excessive sweating |
| SJX-653 | None | Phase II | Hot flashes |
| Talnetant | DB06429 | Phase II | Schizophrenia, irritable colon |
| Osanetant | DB04872 | Phase II | Prostate Adenocarcinoma, Post-menopausal Vasomotor Symptoms |

Data is sourced from: ClinicalTrials.gov(https://clinicaltrials.gov/) and DrugBank(<https://go.drugbank.com/>).
