# Supplementary material for: Noval insights and therapeutic strategies for tumor-induced kidney pathologies
Source: J Exp Clin Cancer Res. 2024 Oct 19;43:289. doi: 10.1186/s13046-024-03205-6 (PMC11490039; doi:10.1186/s13046-024-03205-6)
Supplement: Supplementary file 2 — Supplementary Material 2 [file 13046_2024_3205_MOESM2_ESM.docx]

Dear Dr. Castelli,

Thank you for your email and for the constructive feedback provided by the reviewers regarding our manuscript titled *"Noval Insights and Therapeutic Strategies for Tumor-Induced Kidney Pathologies"* (JECC-D-24-02138). We greatly appreciate the reviewers' insightful comments, which help us to further improve the quality of our work.

We have carefully addressed each of the points raised by the reviewers and made the necessary revisions to the manuscript. Here is a point-by-point response to the reviewers' comments.

**Response to Reviewer #1:**

Thank you for your valuable comments and suggestions. We have carefully reviewed your feedback and revised the manuscript accordingly. Below is our point-by-point response:

1. **Using interspecies conservation to elucidate the role of TACR3 in humans is reasonable, but as the article summarizes, "there are many factors contributing to kidney injury." What mechanisms of kidney injury caused by these factors can be explained by the role of TACR3?**

*Response:* Thanks for your comments. The study by Xu et al. provides insights into how tumor-derived factors, specifically the novel antidiuretic hormone ITPF, lead to renal dysfunction. In Drosophila models, ITPF impairs renal function by targeting the G-protein-coupled receptor (TkR99D), a homolog of the mammalian neurokinin 3 receptor (TACR3). This interaction activates the nitric oxide synthase-cGMP signaling pathway in renal cells, leading to inhibited fluid excretion and contributing to symptoms like bloating and fluid accumulation.

In mammals, TACR3 plays a similar role. Tumor-induced upregulation of TACR3 in renal tissues, particularly in cases of cancer-associated renal dysfunction, activates analogous pathways that impair kidney function. By blocking TACR3 activity in mouse models, renal dysfunction was alleviated, indicating its crucial role in the pathology of cancer-related kidney injuries.

Thus, TACR3 mediates renal injury through its involvement in fluid retention, disruption of renal excretory functions, and possibly inflammatory or immune responses triggered by tumor-related stress. These findings underline the therapeutic potential of TACR3 antagonists in mitigating such injuries in cancer patients.

2. **Is there clinical data supporting TACR3, specifically regarding the relationship between TACR3 and tumor-associated nephropathy?**
   *Response:* Thank you for your insightful question. Unfortunately, there is currently no clinical data directly linking TACR3 to tumor-associated nephropathy. However, we have identified promising preclinical studies that demonstrate the upregulation of TACR3 gene expression in renal tissues under oncological stress (Figure 3A-3E). In particular, our research has examined TACR3 expression across various stages of kidney renal clear cell carcinoma (KIRC) and kidney renal papillary cell carcinoma (KIRP). Through multi-omics analysis, we observed significant differential TACR3 expression between tumor stages, with statistical analysis confirming the importance of this variance (Figure 3F-3G). This highlights the potential of TACR3 as a therapeutic target in these cancers.

However, as both the reviewer's concern and our summary indicate, despite promising preclinical evidence, we emphasize the need for future clinical trials to validate these findings and determine whether TACR3 antagonists can be effectively employed in the treatment of renal carcinomas and tumor-associated nephropathy.

3. **Potential side effects of TACR3 antagonists need attention, as well as their relationship with cancer treatment.**
   *Response:* Thank you for raising this important point. We have included the main side effects of TACR3 antagonists in the revised manuscript, specifically in lines 101 to 111.

Clinical data from the testing of several TACR3 antagonists have highlighted the need for hepatic monitoring due to asymptomatic increases in hepatic transaminases, though bilirubin levels remain unaffected[1]. Additionally, there have been signals regarding small elevations in blood glucose. The most common side effects associated with fezolinetant, a specific TACR3 antagonist, reported in 2.3%–4.3% of participants in a 52-week safety trial, include abdominal pain, diarrhea, insomnia, back pain, hot flashes, and elevated hepatic transaminase levels[2]. It’s also important to note that these side effects were more prevalent compared to placebo. The potential for TACR3 antagonists in cancer treatment, especially for tumor-associated conditions, is promising, but these adverse effects underline the need for careful monitoring and further investigation to ensure safety when used in conjunction with cancer therapies.

1.	Lee, A., *Fezolinetant: First Approval.* Drugs, 2023. **83**(12): p. 1137-1141.

2.	Neal-Perry, G., et al., Safety of Fezolinetant for Vasomotor Symptoms Associated With Menopause: A Randomized Controlled Trial. Obstet Gynecol, 2023. **141**(4): p. 737-747.

4. **The relationship between TACR3 and kidney injury involves the activation of TACR3 receptors in kidney tissue, while in renal cancer, the focus is primarily on the expression of TACR3 in tumor cells. Should there be a focus on the status of TACR3 in normal renal tubular cells, and should more attention be paid to the expression of TACR3 ligands in tumors? This would help in understanding the mechanisms by which TACR3 induces kidney injury in both normal and tumor tissues.**
   *Response:* We sincerely appreciate this insightful suggestion. As depicted in Figure 3E and the accompanying lower panel (Dot plot illustrating TACR3 expression levels across various renal cell types in the normal human kidney; colors denote the intensity of TACR3 expression. Data sourced from Wu and Uchimura et al., Cell Stem Cell, 2018; RBK RID: 14-4KPM.), TACR3 is predominantly expressed in podocytes within the normal kidney. Through the single-cell data presented in Figure 3E, we observe TACR3 expression across a range of renal cell types under normal conditions, as well as notable changes in its expression across different cell types during kidney injury.

Regarding the expression of TACR3 ligands in tumors, as highlighted in lines 58-59 of the manuscript, TACR3 in both humans and mice binds to natural peptide agonists such as Neurokinin B (NKB) and Substance P, along with the synthetic agonist Senktide. However, in Drosophila, as discussed in lines 47-49, TACR3's ligand is ITPF, a novel antidiuretic hormone identified as a crucial factor in cancer-associated renal dysfunction. The homologous ligand of ITPF in humans and mice remains unidentified, and further research is required to determine its presence and function.

Given the high expression of TACR3 and the demonstrated efficacy of TACR3 antagonists in tumors from Xu et al.'s study, it is worth noting that the mammalian TACR3 serves as a homolog of the G-protein-coupled receptor TkR99D in Drosophila. In light of this, we emphasize the potential therapeutic role of TACR3 antagonists, especially in the context of renal dysfunction and cancer.

However, as the reviewer astutely pointed out, it is equally important to further investigate the mechanisms by which TACR3 induces kidney injury in both normal and tumor tissues. Understanding the interaction between TACR3 and its ligands in these pathological conditions is crucial. Further research and clinical trials are essential to fully elucidate the role of TACR3 in cancer-associated renal dysfunction, potentially leading to more targeted and effective therapeutic strategies.

Figure 3E:

5. **What are the connections and differences between TACR3 and NK3R?**
   *Response:* Thank you for your comment. TACR3 is also known as NK3R (neurokinin 3 receptor), and we have now clarified this in the manuscript to prevent any confusion. Both terms refer to the same receptor, and we have ensured consistency throughout the manuscript. The changes have been made on lines 58, 140, and 144.

**Response to Reviewer #2:**
This commentary article by Meng Wang et al summarized recent progress in discovery of TACR3 as a potential therapeutic target for tumor-induced kidney dysfunction. The authors first reported the recent work by Xu et al to identify ITPF in Drosophila and the mammalian TACR3 as a potential therapeutic target in cancer-related renal dysfunction. Then they analyzed the evolutionary trajectory of TACR3 to confirm its importance in biological functions. The authors also performed molecular docking to find the key binding domains of TACR3 for its pharmacological activity, they further conducted a comprehensive analysis of TACR3 expression in various cancer types using publicly available datasets and demonstrated that TACR3 might play important roles in tumorigenesis and cancer progression other that its functions in renal dysfunctions. Overall, the authors did a fantastic job to summarize recent work of TACR3, and performed bioinformatics analysis to help design targeted therapeutics of TACR3 and find other biological functions of TACR3. The manuscript is well written, clear and organized. I suggest the editor to consider publish this work as soon as possible.

*Response:* Thank you very much for your positive feedback and for recognizing the strengths of our manuscript. We are delighted that you found the summary of TACR3 research, including its therapeutic potential and role in cancer-related renal dysfunction, to be well-executed and informative. Your encouraging words motivate us to continue refining and improving our work.

If you require any further information or clarification, please do not hesitate to contact me. We look forward to your feedback.

Thank you once again for your time and consideration.

Best regards,

Chao Zhang, [zhangchao@shsmu.edu.cn](mailto:zhangchao@shsmu.edu.cn)

*Department of Orthopedics and Precision Research Center for Refractory Diseases, Shanghai General Hospital,* *Shanghai Jiao Tong University School of Medicine*
